# Large Language Model Influence on Diagnostic Reasoning: A Randomized Clinical Trial

**DOI:** 10.1001/jamanetworkopen.2024.40969

**Published:** 2024-10-28

**Authors:** Ethan Goh, Robert Gallo, Jason Hom, Eric Strong, Yingjie Weng, Hannah Kerman, Joséphine A. Cool, Zahir Kanjee, Andrew S. Parsons, Neera Ahuja, Eric Horvitz, Daniel Yang, Arnold Milstein, Andrew P. J. Olson, Adam Rodman, Jonathan H. Chen

**Affiliations:** 1 Stanford Center for Biomedical Informatics Research, Stanford University, Stanford, California; 2 Stanford Clinical Excellence Research Center, Stanford University, Stanford, California; 3 Center for Innovation to Implementation, VA Palo Alto Health Care System, Palo Alto, California; 4 Department of Hospital Medicine, Stanford University School of Medicine, Stanford, California; 5 Quantitative Sciences Unit, Stanford University School of Medicine, Stanford, California; 6 Department of Hospital Medicine, Beth Israel Deaconess Medical Center, Boston, Massachusetts; 7 Department of Hospital Medicine, Harvard Medical School, Boston, Massachusetts; 8 Department of Hospital Medicine, School of Medicine, University of Virginia, Charlottesville; 9 Microsoft Corp, Redmond, Washington; 10 Stanford Institute for Human-Centered Artificial Intelligence, Stanford, California; 11 Department of Hospital Medicine, Kaiser Permanente, Oakland, California; 12 Department of Hospital Medicine, University of Minnesota Medical School, Minneapolis; 13 Division of Hospital Medicine, Stanford University, Stanford, California

## Abstract

**Importance:**

Large language models (LLMs) have shown promise in their performance on both multiple-choice and open-ended medical reasoning examinations, but it remains unknown whether the use of such tools improves physician diagnostic reasoning.

**Objective:**

To assess the effect of an LLM on physicians’ diagnostic reasoning compared with conventional resources.

**Design, Setting, and Participants:**

A single-blind randomized clinical trial was conducted from November 29 to December 29, 2023. Using remote video conferencing and in-person participation across multiple academic medical institutions, physicians with training in family medicine, internal medicine, or emergency medicine were recruited.

**Intervention:**

Participants were randomized to either access the LLM in addition to conventional diagnostic resources or conventional resources only, stratified by career stage. Participants were allocated 60 minutes to review up to 6 clinical vignettes.

**Main Outcomes and Measures:**

The primary outcome was performance on a standardized rubric of diagnostic performance based on differential diagnosis accuracy, appropriateness of supporting and opposing factors, and next diagnostic evaluation steps, validated and graded via blinded expert consensus. Secondary outcomes included time spent per case (in seconds) and final diagnosis accuracy. All analyses followed the intention-to-treat principle. A secondary exploratory analysis evaluated the standalone performance of the LLM by comparing the primary outcomes between the LLM alone group and the conventional resource group.

**Results:**

Fifty physicians (26 attendings, 24 residents; median years in practice, 3 [IQR, 2-8]) participated virtually as well as at 1 in-person site. The median diagnostic reasoning score per case was 76% (IQR, 66%-87%) for the LLM group and 74% (IQR, 63%-84%) for the conventional resources-only group, with an adjusted difference of 2 percentage points (95% CI, −4 to 8 percentage points; *P* = .60). The median time spent per case for the LLM group was 519 (IQR, 371-668) seconds, compared with 565 (IQR, 456-788) seconds for the conventional resources group, with a time difference of −82 (95% CI, −195 to 31; *P* = .20) seconds. The LLM alone scored 16 percentage points (95% CI, 2-30 percentage points; *P* = .03) higher than the conventional resources group.

**Conclusions and Relevance:**

In this trial, the availability of an LLM to physicians as a diagnostic aid did not significantly improve clinical reasoning compared with conventional resources. The LLM alone demonstrated higher performance than both physician groups, indicating the need for technology and workforce development to realize the potential of physician-artificial intelligence collaboration in clinical practice.

**Trial Registration:**

ClinicalTrials.gov Identifier: NCT06157944

## Key Points

**Question:**

Does the use of a large language model (LLM) improve diagnostic reasoning performance among physicians in family medicine, internal medicine, or emergency medicine compared with conventional resources?

**Findings:**

In a randomized clinical trial including 50 physicians, the use of an LLM did not significantly enhance diagnostic reasoning performance compared with the availability of only conventional resources.

**Meaning:**

In this study, the use of an LLM did not necessarily enhance diagnostic reasoning of physicians beyond conventional resources; further development is needed to effectively integrate LLMs into clinical practice.

## Teaser

This randomized clinical trial evaluates the diagnostic performance of physicians with use of a large language model compared with conventional resources.

## Introduction

Diagnostic errors are common, contribute to substantial patient harm, and result from a combination of cognitive and systems factors.^1,2,3,4,5^ Effective interventions to improve diagnostic performance and reduce diagnostic errors will need to focus on both systems factors and cognitive factors, often referred to as clinical reasoning. Strategies that have been advanced to improve clinical reasoning include a variety of educational, reflective, and team-based practices, as well as clinical decision support tools.^6^ The impact of these interventions has been limited, and even the most useful methods, such as reflective practice, are difficult to integrate clinically at scale.^7,8^ Artificial intelligence (AI) technologies have long been pursued as promising tools for assisting physicians with diagnostic reasoning.

Large language models (LLMs)—machine learning systems that produce humanlike responses from written language—have shown the ability to solve complex cases, exhibit humanlike clinical reasoning, take patient histories, and display empathetic communication.^9,10,11,12,13,14^ Due to their generalizable nature, LLMs are actively being integrated into multiple health care settings.^15,16,17,18,19,20^ Despite the impressive performance of these emerging technologies in benchmarking tasks, current integrations of LLMs require human participation, with the LLM augmenting, rather than replacing, human expertise and oversight.^21^ Understanding the implications of deploying these systems in patient care with limited workforce training and integration requires human-computer user studies with richer measures of diagnostic reasoning.

We performed a randomized clinical trial to compare the diagnostic reasoning performance of physicians using a commercial LLM AI chatbot (ChatGPT Plus [GPT-4]; OpenAI) compared with conventional diagnostic resources (eg, UpToDate, Google). Many studies of diagnostic performance only assess shallow measures of accuracy without attention to the quality of the diagnostic process used to arrive at that diagnosis. To develop a deeper assessment of how new tools affect physician reasoning, we further adapted structured reflection—a measure of factors contributing to a diagnostic decision—as a novel assessment tool of the diagnostic process.^22^

## Methods

This study was reviewed and determined to be exempt from approval by institutional review boards at Stanford University, Beth Israel Deaconess Medical Center, and the University of Virginia. Informed consent was obtained prior to enrollment and randomization. Resident participants were offered $100 and attending participants were offered up to $200 for completing the study. This study follows the Consolidated Standards of Reporting Trials (CONSORT) reporting guideline for randomized clinical trials. The study protocol is available in Supplement 1.

We recruited attending and resident physicians with training in a general medical specialty (internal medicine, family medicine, or emergency medicine) through email lists at Stanford University, Beth Israel Deaconess Medical Center, and the University of Virginia. Small groups of participants were proctored by study coordinators either remotely or at an in-person computer laboratory. Sessions lasted for 1 hour. The participant flow is depicted in the Figure. A visual iteration is presented in eFigure 2 in Supplement 2.

**Figure.**
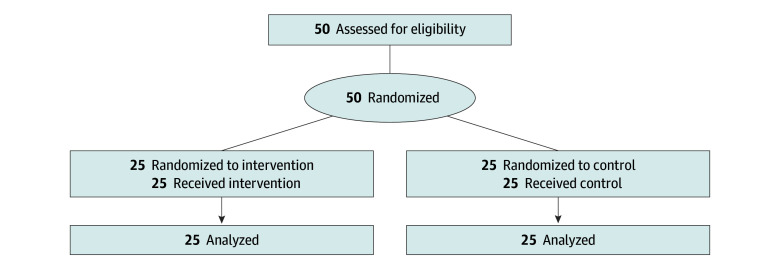
Participant Flowchart

### Clinical Vignettes

Clinical vignettes were adapted from a landmark study that set the standard for the evaluation of computer-based diagnostic systems.^23^ All cases in this study were based on actual patients and included information available on initial diagnostic evaluation, such as history, physical examination, and laboratory test results. The cases have never been publicly released to protect the validity of the test materials for future use, and therefore are excluded from training data of the LLM. A representative example is included in eTable 1 in Supplement 2. We used the nominal group technique to select a cross-section of cases; 4 physician authors (E.G., J.A.C., A.P.J.O., and J.H.C.) met to agree on case selection guidelines including preference for a broad range of pathologic settings, avoiding simplistic cases with limited plausible diagnoses, and excluding exceedingly rare cases.^24^ Each member independently reviewed at least 50 of the 105 available cases to identify a minimum of 10 cases that satisfied selection guidelines. After individual ratings, the group convened again to come to a consensus on a prioritized list of cases to consider. In pilot tests, participants completed a maximum of 6 cases in 1 hour, leading us to select 6 final cases for this study. Cases were edited to modernize laboratory data reporting conventions and to replace pathognomic phrases (eg, livedo reticularis) with general descriptions (eg, purple, red, lacy rash).

A common, but limited, evaluation benchmark in clinical decision support diagnostic studies is accuracy of differential diagnosis. While we assessed overall differential diagnosis accuracy as a secondary outcome similar to prior studies, the complex phenomena of human-computer interactions warrant richer evaluations of diagnostic reasoning skills. We therefore chose to develop an assessment from the clinical reasoning literature: structured reflection.^25^

Structured reflection aims to improve the process by which physicians consider reasonable diagnoses and clinical features that support or oppose their diagnoses, similar to how physicians may explain their reasoning in the assessment and plan component of clinical notes.^25,26^ We adapted a structured reflection grid (eTable 1 in Supplement 2) with participants providing free-text responses on their top 3 differential diagnoses, the factors in the case that favor or oppose each of their 3 diagnoses, their final most likely diagnosis, and up to 3 next steps (eg, diagnostic tests) they would use to further evaluate the patient.

### Assessing Performance

We built on previous studies of structured reflection by scoring the grid itself, not just final diagnosis accuracy. For each case, we assigned up to 1 point for each plausible diagnosis. Findings supporting each diagnosis and findings opposing the diagnosis were also graded based on correctness, with 0 points for incorrect or absent answers, 1 point for partially correct, and 2 points for completely correct responses. The final diagnosis was graded as 2 points for the most correct diagnosis and 1 point for a plausible diagnosis or a correct diagnosis that was not specific enough compared with the most correct final diagnosis. The participants then were instructed to describe up to 3 next steps to further evaluate the patient, with 0 points awarded for an incorrect response, 1 point awarded for a partially correct response, and 2 points awarded for a completely correct response (eTable 2 in Supplement 2). While incorrect differential diagnoses were not awarded points, appropriate reasoning based on those diagnoses were not penalized. Raters were blinded to participant group assignments.

### Study Design

We used a randomized single-blind study design with stratified randomization. Participants were randomized to use the LLM interface (intervention group) or conventional resources (control group). They were given access to study accounts for the LLM, and transcripts from their use were saved. Both groups were instructed to access any conventional resources they normally use for clinical care, but the control group was explicitly instructed not to use LLMs. Participants had 1 hour to complete as many of the 6 diagnostic cases as they could, with instructions to prioritize quality of their responses over completing all cases.

The study was conducted using a survey tool (Qualtrics), with cases presented in random order for each participant. In a secondary analysis, we included a comparison arm using the LLM alone to answer the cases. Using established principles of prompt design, we iteratively developed a 0-shot prompt; the same language was used along with the clinical vignette questions for each case.^27^ The researcher physician inputting prompts to the model did not alter model responses. eTable 4 in Supplement 2 gives an example prompt. These prompts were run 3 times in separate sessions, and the results from each run were included for blinded grading alongside the human outputs before unblinding or data analysis.

### Assessment Tool Validation

To establish validity, we collected 2 sets of pilot data with 13 participants not included in the final study. This included a total of 65 cases completed, based on a sampling of multiple case vignettes, including the 6 used in the final study. The 3 primary scorers (J.H, A.P.J.O., and A.R.), all board-certified physicians with experience in the evaluation of clinical reasoning at the postgraduate medical level, graded these independently to assess consistency. Based on iterative feedback from both graders and pilot participants, as well as grader concordance, the study case vignettes were selected and rubrics were further refined before data were collected for the final study. After data collection, each case was graded independently by 2 scorers who were blinded to the assigned treatment group. Disagreement between scorers was predefined as a difference of more than 10% of the final score. eTable 5 in Supplement 2 gives variance by subcomponents. When scorers disagreed, they met to discuss differences in their assessments and seek consensus. We designed scoring to intentionally acknowledge ambiguity in diagnostic processes, allowing for multiple variations of correct answers determined by scorer consensus. Final diagnosis scoring was adjudicated by 2 scorers to obtain agreement for the secondary outcome of diagnostic accuracy. We calculated a weighted Cohen κ value to show concordance in grading and Cronbach α value to determine the internal reliability of this measure.^28,29^

### Study Outcomes

Our primary outcome was the final score as a percentage across all components of the structured reflection tool. Secondary outcomes were time spent per case (in seconds) and final diagnosis accuracy. Final diagnosis was treated as an ordinal outcome with 3 groups (incorrect, partially correct, and most correct). Since the difference between the most correct response and partially correct responses may not be clinically meaningful, we additionally analyzed the outcome as binary (incorrect compared with at least partially correct).

### Statistical Analysis

The target sample size of 50 participants was prespecified based on a power analysis using 2 validation sets of data, scored before study enrollment. Using PASS 2023, version 23.0.2 software (NCSS LLC), our power analysis showed more than 80% power to detect an 8% score difference with 200 to 250 cases completed (4 to 5 cases per participant) with a 2-sided α value of .05. We used a mixed-effects model suitable for cluster-randomized designs, with an intraclass correlation coefficient ranging from 0.05 to 0.15 and an SD of 16.2%.

All analyses followed the intention-to-treat principle and were conducted at the case level, clustered by the participant. Linear mixed-effects models were applied to assess the difference in the primary outcome of diagnostic performance and the secondary outcome of time spent per completed case, with normality assumptions verified. Ordinal and logistic mixed-effects models were used for comparisons of other secondary outcomes including ordinal and binary final diagnosis accuracy. A random effect for the participant was included in the models to account for the potential correlation between cases for a participant. Additionally, a random effect for cases was included to account for any potential variability in difficulty across cases. Family-wise type I error (α) was controlled at .05 for the primary outcome of diagnostic performance considered as a continuous variable. Analysis of the secondary outcomes was exploratory without adjusting for multiple comparisons. A preplanned sensitivity analysis evaluated the effect of including incomplete cases on the primary outcome. Subgroup analyses were conducted based on training status and experience with the LLM product used. In a secondary analysis, cases completed by the LLM alone were treated as a third group, with cases clustered in a nested structure of 3 attempts under a single participant. These were compared with cases from real participants with each case considered as a single attempt under a single participant using a similar nested structure.

All statistical analysis was performed using R, version 4.3.2 (R Foundation for Statistical Computing). Further details regarding the trial protocol and statistical analysis plan are provided in Supplement 1.

## Results

Fifty US-licensed physicians were recruited and participated (26 attendings, 24 residents) from November 29 to December 29, 2023; of these, 39 (78%) participated in virtual encounters and 11 (22%) were in-person. Median years in practice was 3 (IQR, 2-8). Further information on participants is included in Table 1.

**Table 1.** Baseline Participant Characteristics

Participant characteristic	Participants, No. (%)		
	Overall (N = 50)	Physicians plus LLM (n = 25)	Physicians plus conventional resources (n = 25)
Career stage			
Attending	26 (52)	13 (52)	13 (52)
Resident	24 (48)	12 (48)	12 (48)
Specialty			
Internal medicine	44 (88)	22 (88)	22 (88)
Family medicine	1 (2)	1 (4)	0
Emergency medicine	5 (10)	2 (8)	3 (12)
Years in practice, median (IQR)	3 (2-8)	3 (2-7)	3 (2-9)
LLM experience			
I’ve never used it before	8 (16)	5 (20)	3 (12)
I’ve used it once ever	6 (12)	4 (16)	2 (8)
I use it rarely (less than once per month)	15 (30)	7 (28)	8 (32)
I use it occasionally (more than once per month but less than weekly)	13 (26)	6 (24)	7 (28)
I use it frequently (weekly or more)	8 (16)	3 (12)	5 (20)

Abbreviation: LLM, large language model.

### Primary Outcome: Diagnostic Performance

A total of 244 cases were completed by all participants (125 cases in LLM group, 119 cases in control group). The median number of completed cases per participant was 5 (IQR, 4-6). Analysis of the transcripts showed that 100% (22 of 22) of physicians randomized to use the LLM did so; 3 transcripts were lost due to technical issues and not included. The median score per case was 76% (IQR, 66%-87%) for the LLM group and 74% (IQR, 63%-84%) for the control group. The mixed-effects model showed a difference of 2 percentage points (95% CI, −4 to 8 percentage points; *P* = .60) between the LLM and control groups, as presented in Table 2. A sensitivity analysis including all cases, complete and incomplete, showed a similar result with a difference of 2 percentage points (95% CI, −4 to 8 percentage points; *P* = .50) between the LLM and control group. The distribution of diagnostic performance scores by group is given in eFigure 1 in Supplement 2.

**Table 2.** Diagnostic Performance Outcomes

Group	Median (IQR), %		Difference (95% CI), percentage points^a^	*P* value
	Physicians plus LLM	Physicians plus conventional resources		
All participants	76 (66 to 87)	74 (63 to 84)	2 (−4 to 8)	.60
Level of training				
Attending	79 (63 to 87)	75 (61 to 87)	0.5 (−9 to 1)	.92
Resident	76 (68 to 84)	74 (63 to 84)	3 (−6 to 11)	.50
LLM experience				
Less than monthly	76 (63 to 84)	76 (63 to 87)	−0.5 (−8 to 7)	.90
More than monthly	79 (68 to 90)	74 (63 to 84)	5 (−7 to 16)	.40

Abbreviation: LLM, large language model.

^a^ Differences between groups are reported from the multilevel analysis accounting for clustering of cases by participant.

### Secondary Outcomes

Median time spent per case was 519 (IQR, 371-668) seconds for the LLM group and 565 (IQR, 456-788) seconds for the control group (Table 3). The linear mixed-effects model resulted in an adjusted difference of −82 seconds (95% CI, −195 to 31 seconds; *P* = .20).

**Table 3.** Time Spent per Case

Group	Median (IQR) time, s		Difference (95% CI)^a^	*P* value
	Physicians plus LLM	Physicians plus conventional resources		
All participants	519 (371 to 668)	565 (456 to 788)	−82 (−195 to 31)	.15
Level of training				
Attending	533 (389 to 672)	563 (435 to 778)	−73 (−204 to 58)	.26
Resident	478 (356 to 654)	565 (458 to 800)	−76 (−284 to 131)	.45
LLM experience				
Less than monthly	556 (415 to 742)	572 (474 to 778)	−46 (−219 to 127)	.59
More than monthly	462 (305 to 627)	556 (427 to 810)	−140 (−294 to 13)	.07

Abbreviation: LLM, large language model.

^a^ Differences between groups are reported from the multilevel analysis accounting for clustering of cases by participant.

Accuracy of the final diagnosis (eTable 3 in Supplement 2) using the ordinal scale showed the LLM intervention group had 1.4 times higher odds (95% CI, 0.7-2.8; *P* = .39) of a correct diagnosis than the control group. In assessing the accuracy of final diagnoses, treating them as binary (correct vs incorrect) variables did not qualitatively change the results (odds ratio, 1.9; 95% CI, 0.9-4.0; *P* = .10).

### Subgroup Analyses

Table 2 and Table 3 include the analyses by subgroups, including level of training and level of experience with the LLM. Subgroup analyses were qualitatively similar to the analyses for the whole cohort.

### LLM Alone

In the 3 runs of the LLM alone, the median score per case was 92% (IQR, 82%-97%). Comparing LLM alone with the control group found an absolute score difference of 16 percentage points (95% CI, 2-30 percentage points; *P* = .03) favoring the LLM alone.

### Assessment Tool Validation

The weighted Cohen κ value between all 3 graders was 0.66, indicating substantial agreement within the expected range for diagnostic performance studies.^30^ The overall Cronbach α value was 0.64. The variances of individual sections of the structured reflection rubric are presented in eTable 5 in Supplement 2. After removing the final diagnosis, which had the highest variance, the Cronbach α value was 0.67.

## Discussion

This randomized clinical trial found that physician use of a commercially available LLM chatbot did not improve diagnostic reasoning on challenging clinical cases, despite the LLM alone significantly outperforming physician participants. The results were similar across subgroups of different training levels and experience with the chatbot. These results suggest that access alone to LLMs will not improve overall physician diagnostic reasoning in practice. These findings are particularly relevant now that many health systems offer Health Insurance Portability and Accountability Act–compliant chatbots that physicians can use in clinical settings, often with no to minimal training on how to use these tools.^15,17,18,19^

Our data did not confirm any differences in time spent solving cases. With wide variability observed in time to complete cases, future studies with substantially larger sample sizes would be necessary to evaluate whether physicians with experience using LLMs spend less time on diagnostic reasoning.

An unexpected secondary result was that the LLM alone performed significantly better than both groups of humans, similar to a recent study with different LLM technology.^31^ This may be explained by the sensitivity of LLM output to prompt formulation.^32^ There are numerous frameworks for prompting LLMs and an emerging consensus on prompting strategies, many of which focus on providing details on the task, context, and instructions; our prompt was iteratively developed using these frameworks. Training clinicians in best prompting practices may improve physician performance with LLMs. Alternatively, organizations could invest in predefined prompting for diagnostic decision support integrated into clinical workflows and documentation, enabling synergy between the tools and clinicians. Prior studies on AI systems show disparate effects depending on the component of the diagnostic process they are used in.^33,34^ Given the conversational nature of chatbots, changes in how the LLM interacts with humans, for example by specifically pointing out features that do not fit the differential diagnosis, might improve diagnostic and reflective performance.^35,36^ More generally, we see opportunity with deliberate consideration and redesign of medical education and practice frameworks that adapt to disruptive emerging technologies and enable the best use of computer and human resources to deliver optimal medical care.

Results of this study should not be interpreted to indicate that LLMs should be used for diagnosis autonomously without physician oversight. The clinical case vignettes were curated and summarized by human clinicians, a pragmatic and common approach to isolate the diagnostic reasoning process, but this does not capture competence in many other areas important to clinical reasoning, including patient interviewing and data collection.^37^ Furthermore, this study was acontextual, and clinicians’ understanding of the clinical environment is fundamental for high-quality decision-making. While early studies show that LLMs might effectively collect and summarize patient information, these capabilities need to be studied more thoroughly.^12,16^ Additionally, improvement in rubric scoring here represents an important signal of clinical reasoning, but broader clinical trials are necessary to assess for meaningful differences in downstream clinical impact.

This study developed a measure based on structured reflection, inspired by research on physician cognition.^38^ Scoring the adapted structured reflection tool as a primary outcome represents a novel contribution of this study to offer a richer evaluation framework of diagnostic reasoning skills. This assessment tool demonstrated substantial agreement between graders and internal reliability similar or superior to other measures used in the assessment of reasoning.^39,40,41,42^ This advances the field beyond early LLM research, which has focused on benchmarks with limited clinical utility, such as multiple-choice question banks used for medical licensing or curated case vignettes of diseases rarely seen in clinical practice, such as clinicopathologic case conferences.^11,43^ While having obvious advantages in ease of measurement, these tasks are not consistent with clinical reasoning in practice. As AI research progresses and nears clinical integration, it will become even more important to reliably measure diagnostic performance using the most realistic and clinically relevant evaluation methods and metrics.

### Limitations

This trial has limitations. We focused our investigation around a single LLM, given its commercial availability and integration into clinical practice.^15,17,18,19^ Multiple alternative LLM systems are rapidly emerging, although the one studied currently remains among the most performant tools for the applications studied.^44,45^ Participants were given access to the chatbot without explicit training in prompt engineering techniques that could have improved the quality of their interactions with the system; however, this is consistent with current integrations and thus requires this representative evaluation.^15,17,18,19^ Furthermore, even though all of the physicians in the LLM arm at least tried to use the system based on chat logs, they were not forced to use the system in any consistent way. This was a purposeful design to better reflect an effectiveness evaluation in the clinical practice setting.

No sample of clinical vignettes can comprehensively cover the variety of cases in the field of medicine. Our study included 6 cases that could feasibly be completed within a single study session while remaining comparable to standard practices in national licensing and objective structured clinical examinations to use a small, but broad sample of clinical cases.^6,46,47,48,49^ This is not meant to comprehensively assess a participant’s knowledge, but rather to evaluate their general clinical reasoning across a set of cases. To maximize a range of coverage, we deliberately selected cases to capture a broad and relevant cross-section of disciplines and a range of clinical problems.

## Conclusions

The availability of an LLM as a diagnostic aid did not improve physician performance compared with conventional resources in a diagnostic reasoning randomized clinical trial. The LLM alone outperformed physicians even when the LLM was available to them, indicating that further development in human-computer interactions is needed to realize the potential of AI in clinical decision support systems.

## Notes

Supplement 1.
Study Protocol

Supplement 2. **eTable 1.** Diagnostic Case #1 Vignette and Questions **eTable 2.** Structured-Reflection Rubric for Diagnostic Case #1, With Example of high-Scoring vs Low-Scoring Example Responses **eFigure 1.** Distribution of Diagnostic Performance Scores of Physician + GPT-4 vs Physician + Conventional Resources Only **eTable 3.** GPT-4 Performance **eTable 4.** GPT Prompt and Responses for Diagnostic Case 1 **eTable 5.** Assessment Tool Validation **eFigure 2.** Visual Flow Diagram

Supplement 3.
Data Sharing Statement
