# Supplementary material for: Large Language Model Influence on Diagnostic Reasoning: A Randomized Clinical Trial
Source: JAMA Netw Open. 2024 Oct 28;7(10):e2440969. doi: 10.1001/jamanetworkopen.2024.40969 (PMC11519755; doi:10.1001/jamanetworkopen.2024.40969)
Supplement: Supplement 1. — Study Protocol [file jamanetwopen-e2440969-s001.pdf]

Study Description

Brief Summary:

This study will evaluate the effect of providing access to GPT-4, a large language model, compared to traditional diagnostic decision support tools on performance on case-based diagnostic reasoning tasks.

Condition or disease: Diagnosis

Intervention/treatment: Other: GPT-4

Phase: Not Applicable

Detailed Description:

Artificial intelligence (AI) technologies, specifically advanced large language models like OpenAI's ChatGPT, have the potential to improve medical decision-making. Although ChatGPT-4 was not developed for its use in medical-specific applications, it has demonstrated promise in various healthcare contexts, including medical note-writing, addressing patient inquiries, and facilitating medical consultation. However, little is known about how ChatGPT augments the clinical reasoning abilities of clinicians.

Clinical reasoning is a complex process involving pattern recognition, knowledge application, and probabilistic reasoning. Integrating AI tools like ChatGPT-4 into physician workflows could potentially help reduce clinician workload and decrease the likelihood of missed diagnoses. However, ChatGPT-4 was not developed for the purpose of clinical reasoning nor has it been validated for this purpose. Further, it may be subject to disinformation, including convincing confabulations that may mislead clinicians. If clinicians misuse this tool, it may not improve diagnostic reasoning and could even cause harm. Therefore, it is important to study how clinicians use large language models to augment clinical reasoning prior to routine incorporation into patient care.

In this study, we will randomize participants to answer diagnostic cases with or without access to ChatGPT-4. The participants will be asked to give three differential diagnoses for each case, with supporting and opposing findings for each diagnosis. Additionally, they will be asked to provide their top diagnosis along with next diagnostic steps. Answers will be graded by independent reviewers blinded to treatment assignment.

Study Design

Study Type: Interventional (Clinical Trial)

Actual Enrollment: 50 participants

Allocation: Block randomization with block sizes of 4, 6, and 8; stratified by resident and attending physicians. Randomization list was created by Yingjie Weng using the Sealed Envelope program.

Intervention Model: Parallel Assignment

Intervention Model Description: The trial will be designed as a cluster randomized, two-arm, single-blind parallel group study.

Masking: Single (Outcomes Assessor)

Masking Description: The grading of responses will be performed by assessors blinded to participant identity and treatment assignment.

Primary Purpose: Diagnostic

Official Title: Diagnostic Reasoning With Large Language Model Chat Bots

Actual Study Start Date: November 29, 2023

Actual Primary Completion Date: December 30, 2023

Actual Study Completion Date: December 30, 2023

Arms and Interventions

**Arm**	**Intervention/treatment**
Active Comparator: GPT-4<br><br>Group will be given access to GPT-4.	Other: GPT-4<br><br>OpenAI's GPT-4 large language model with chat interface.
No Intervention: Usual resources<br><br>Group will not be given access to GPT-4 but will be encouraged to use any resources they wish besides large language models (UpToDate, Dynamed, google, etc).	

Outcome Measures

Primary Outcome Measures:

Diagnostic reasoning [Time Frame: During evaluation]

The primary outcome will be the percent correct (range: 0 to 100) for each case. For each case, participants will be asked for three top diagnoses and findings from the case that support that diagnosis and oppose that diagnosis. Participants will receive 1 point for each plausible diagnosis. Findings supporting the diagnosis and findings opposing the diagnosis will also be graded based on correctness, with 1 point for partially correct and 2 points for completely correct responses. Participants will then be asked to name their top diagnosis, earning one point for a reasonable response and two points for the most correct response. Finally participants will be asked to name up to 3 next steps to further evaluate the patient with one point awarded for a partially correct response and two points for a completely correct response. The primary outcome will be compared on the case-level by the randomized groups.

Secondary Outcome Measures:

Time Spent on Diagnosis [Time Frame: During evaluation]

We will compare how much time (in minutes) participants spend per case between the two study arms.

Eligibility Criteria

Ages Eligible for Study:	Child, Adult, Older Adult
Sexes Eligible for Study:	All
Accepts Healthy Volunteers:	Yes

Criteria

Inclusion Criteria:

Participants must be licensed physicians and have completed at least post-graduate year 2 (PGY2) of medical training.

Training in Internal medicine, family medicine, or emergency medicine.

Exclusion Criteria:

Not currently practicing clinically.

Power Analysis:

With a total of 50 participants enrolled, 25 participants per group, we will have >80% power to detect a difference of the score as low as 8%, when comparing AI group to non-AI group using a mixed-effect model in a cluster-randomized design. This is based on the assumptions that we will have on average 4 cases completed per participant, standard deviation of score as 16.2%, ICC in the range from 0.05 to 0.15, and a type I error of 0.05, according to our preliminary data. Power analysis was performed on PASS software.

Statistical Analysis:

Descriptive analysis will be performed by comparing participant characteristics by the AI and non-AI group. Frequencies and proportions will be reported for categorical variables, while mean and standard deviations will be reported for continuous variables. We will also report the median and interquartile range for continuous variables that are not normally distributed. Bland-Altman plot will be generated to identify potential outliers of the scores where discrepancy between the two graders are outside the normal range.

The analyses of the primary outcome will be at the case level, clustered by the participant under the intention-to-treat design. In the primary analysis, cases with completed responses will be included. We will first describe the means and standard deviations of scores that will be standardized to 0-100 as well as median and interquartile range of time spent on each case, in the overall cohort and by the GPT-4

group compared to the conventional resources only group. Box plot will be generated for visualization. To assess the impact of incorporating GPT-4 in the exam, we will further apply generalized mixed-effect models with a random-effect for the participant to account for the potential correlation between cases for a participant, as well as a random effect for cases to account for any potential variability in difficulty across cases. We will consider the following sensitivity analysis:

1) Include incomplete cases on the primary outcome.
2) Include past experience in GPT use as a covariate for adjustment
3) Instead of taking the mean of the scores of the two graders, we plan to randomly select one score from the two graders of each question that are discrepant and rerun the analysis. We will repeat this process multiple times to ensure consistency.

Subgroup analyses will be conducted in a similar fashion, stratified by training status and experience with ChatGPT. Scores for each question of the cases will be also compared descriptively by the two randomized groups.

In a secondary exploratory analysis, cases completed by GPT-4 alone will be treated as a third group with cases clustered in a nested structure of 3 attempts under a single participant. Using a generalized mixed-effect model, these cases will be compared to cases from real participants with each case considered as a single attempt under a single participant using a similar nested structure. Missing data is not expected given our study design. If it occurs, multiple imputation will be considered under the intention-to-treat design for the primary analysis.

All statistical analysis was performed using R statistical programming languages, v4.3.2 (R Foundation for Statistical Computing, Vienna, Austria). Statistical significance was based on a p value <0.05.

Contacts and Locations

Locations

United States, California

Stanford University

Palo Alto, California, United States, 94304

Sponsors and Collaborators

Stanford University

Beth Israel Deaconess Medical Center

University of Minnesota

Investigators

Principal Investigator: Jonathan H Chen, MD, PhD Stanford University

Principal Investigator: Adam Rodman, MD Beth Israel Deaconess Medical Center

Principal Investigator: Andrew Olson, MD University of Minnesota
