# Supplementary material for: Large Language Model Influence on Diagnostic Reasoning: A Randomized Clinical Trial
Source: JAMA Netw Open. 2024 Oct 28;7(10):e2440969. doi: 10.1001/jamanetworkopen.2024.40969 (PMC11519755; doi:10.1001/jamanetworkopen.2024.40969)
Supplement: Supplement 3. — Data Sharing Statement [file jamanetwopen-e2440969-s003.pdf]

Data Sharing Statement

Goh. Large Language Model Influence on Diagnostic Reasoning. *JAMA Netw Open*. Published October 28, 2024. doi:10.1001/jamanetworkopen.2024.40969

Data
Additional Information: Trial Registration ClinicalTrials.gov Identifier: NCT06157944; https://classic.clinicaltrials.gov/ct2/show/NCT06157944
Data available: No

Additional Information
Explanation for why data not available: Example case vignettes, questions, and grading are included in the supplement. GPT-4 transcript chat logs, raw score table, and individual survey responses are available upon request.
